# Pediatric Liver Transplantation Program at the Instituto da Criança do Hospital das Clínicas da Faculdade de Medicina da Universidade de São Paulo

**DOI:** 10.6061/clinics/2016(04)01

**Published:** 2016-04

**Authors:** Ana Cristina Aoun Tannuri, Uenis Tannuri

**Affiliations:** Faculdade de Medicina da Universidade de São Paulo, Instituto da Criança, Unidade de Transplante Hepático, São Paulo/SP, Brazil

The advent of liver transplantation provided a treatment option for several pediatric terminal liver diseases. In September 1989, we performed the first pediatric liver transplantation in a child with biliary atresia at the Instituto da Criança do Hospital das Clínicas da Faculdade de Medicina da Universidade de São Paulo after an extensive program of basic science and animal training. Thanks to institutional efforts and the dedication of a multi-disciplinary team composed of doctors, nurses, hospital administrators, pharmacists, physiotherapists, nutritionists, social workers and psychologists, it was possible for us to create and maintain a Brazilian pediatric liver transplantation program that is comparable to those of the world's best centers. From 1989 to the present, we have performed 680 liver transplantations in children with different terminal liver diseases, including the most severe cases of fulminant hepatic failure.

However, the scarcity of suitable deceased donors has been a great problem for all liver transplantation programs, particularly in the pediatric area because the need to reduce the volume of liver grafts and the relative severity of the clinical conditions of our patients make the procurement of deceased donors even more difficult and selective. The positions of liver recipient candidates on the waiting list (a single list for adults and children) were based on chronologic criteria until June of 2006 and the mean time from inclusion on the list until transplantation was three years. Consequently, waiting-list mortality (affecting more than 50% of patients) was an important problem that had yet to be solved. From that time until the present, despite the introduction of the Pediatric End-stage Liver Disease (PELD)/Model for End-stage Liver Disease (MELD) criteria for the inclusion and classification of patients based on the severity of their hepatic disease and clinical conditions, the waiting-list mortality rate has remained persistently high.

In this context, we began a program of living donor transplantation and the first procedure was performed in 1998. To date, careful selection and preparation of potential living donors have allowed us to perform 280 procedures, with a low morbidity rate and no donor complications or deaths 1. Resection of the left lateral segment or the left lobe (which provides sufficient liver mass for pediatric recipients) is a procedure that is relatively safe for the donors 2. Our center's surgical team includes only pediatric surgeons, with extensive experience in pediatric liver surgery and liver transplantation, including experience with adolescents, which allows us to perform the donor hepatectomies without the participation of adult surgeons. We emphasize that the donor and recipient procedures are performed in adjoining rooms, with full synchronization, in an exclusively pediatric hospital that has undergone logistical and structural adjustments to allow for the procedures in the adults to be performed safely and comfortably. Additionally, in the early postoperative period, the donor (in general, a mother or father) and the recipient can stay together in the same hospital. Finally, another advantage is that the team of pediatric surgeons can perform adequate early and late follow-ups of all donors.

Regarding the benefits of living donor transplantation for the recipients, the advantages of good-quality grafts outweigh the technical difficulties related to the smaller caliber of the vascular anastomoses and to biliary reconstruction 3. Thus, despite the introduction of MELD and PELD scores to determine waiting-list positions, living donor liver transplantation maintains a crucial role in our program because children can be treated earlier and receive extensively evaluated grafts with optimal function that have been exposed to minimal cold ischemia.

However, liver transplantation is a complex procedure, and as such, it is still associated with several postoperative complications. Surgical complications may involve the vascular (hepatic artery, portal vein and hepatic vein) and biliary structures. In this context, we introduced the utilization of simplified arterial microsuture techniques, which make the performance of arterial anastomoses much easier and more practical 4 and the precise utilization of arterial grafts 5, which allows for progressively better results in terms of arterial complications. Moreover, the utilization of a Rex shunt (i.e., a bypass between the superior mesenteric vein and the portal vein left branch) in cases of portal thrombosis 6 and technical modification of the hepatic vein anastomosis procedure, which involves the creation of a wide opening in the anterior wall of the inferior vena cava 7, have been responsible for a decrease in the rate of venous complications.

However, biliary complications and size mismatches remain unsolved problems and are thus the subjects of important experimental studies. A weaning rat model of selective biliary obstruction was developed in our laboratory, with the aim of improving our understanding of biliary cirrhosis in cases of intrahepatic segmental biliary stenosis 8,9.

Moreover, the consequences of engrafting liver grafts that are disproportionately large relative to the recipients' size, i.e., cases of infant recipients who are less than 5 kg in size, have not been completely elucidated. Therefore, we created a new swine “large-for-size” liver transplantation model 10 and many studies that have tentatively clarified the molecular and flowmetric mechanisms of lesions in this situation 11 as well as therapeutic measures to attenuate such lesions have been reported 11.

Finally, the immunologic mechanisms involved in the humoral and cellular rejection of solid organ transplants remain a matter of continuing research in the literature. Despite all of the advances in immunosuppressive regimens and the development of accurate drugs with fewer collateral effects, acute and chronic rejections remain dreaded complications of pediatric liver transplantation. The diagnoses of acute and chronic rejection are based on histopathological findings, and portal inflammation and centrilobular necrosis are characteristics of acute rejection. Moreover, biliary duct paucity in the portal space and foam cell arteriopathy are the main aspects of chronic rejection 12. Regarding acute cellular rejection, treatment and management are relatively standardized and most cases exhibit good responses to corticosteroid bolus treatments as well as tacrolimus level adjustment 13. The treatment and evolution of pediatric patients with chronic rejection are not well characterized, as few published studies are present in the literature.

Indeed, the mechanisms of chronic rejection are not completely known and the progressive biliary duct paucity may not respond to increased immunosuppression, which can lead to severe liver dysfunction, a need for retransplantation or death, as discussed in the paper by Tannuri et al. in the current issue of Clinics. Finally, the early histological findings and histological aspects that are suggestive of irreversibility must be defined, and prognostic factors after chronic rejection is diagnosed must be identified, although certain investigations have previously tried to identify the risk factors for the development of chronic rejection following liver transplantation in pediatric 14 and adult 15,16 populations.

In conclusion, liver transplantation is a relatively new procedure that allows for the survival of many infants and children who otherwise would die. However, this procedure also results in a number of conditions and complications that have never been previously recognized and treated. Therefore, much remains to be studied and accomplished, particularly in the field of immunology.
